# The Effect of a Mobile App (eMOM) on Self-Discovery and Psychological Factors in Persons With Gestational Diabetes: Mixed Methods Study

**DOI:** 10.2196/60855

**Published:** 2025-06-04

**Authors:** Sini Määttänen, Saila Koivusalo, Hanna Ylinen, Seppo Heinonen, Mikko Kytö

**Affiliations:** 1 Faculty of Medicine University of Helsinki Helsinki Finland; 2 Department of Obstetrics and Gynecology Helsinki University Hospital University of Helsinki Helsinki Finland; 3 Shared Group Services Helsinki University Hospital Helsinki Finland; 4 Faculty of Sport and Health Sciences University of Jyväskylä Jyväskylä Finland; 5 Development and Strategy Unit Helsinki University Hospital Helsinki Finland; 6 Department of Computer Science University of Helsinki Helsinki Finland

**Keywords:** self-discovery, self-management, motivation, self-determination theory, self-tracking, gestational diabetes, mobile apps, mixed methods study, diabetes, support, women, pregnancy, maternal, apps, glucose, nutrition, physical activity, psychological, well-being, interview, questionnaire, usability, depression, stress

## Abstract

**Background:**

Gestational diabetes is a type of diabetes that develops during pregnancy and increases the risk of developing type 2 diabetes later in life. The rising prevalence of gestational diabetes mellitus (GDM) highlights the need for more comprehensive treatment strategies, with a particular emphasis on supporting maternal self-management. We showed recently that a mobile app, eMOM, where glucose, nutrition, and physical activity are combined within a single app, significantly improves multiple clinical outcomes among persons with gestational diabetes.

**Objective:**

This study aims to explore the effects of the eMOM on maternal self-discovery and learning, autonomous motivation to manage GDM, and psychological well-being. Additionally, we examine the correlation between improved maternal clinical outcomes and change in autonomous motivation. We also assess the acceptance and usability of the eMOM app.

**Methods:**

Building upon the original randomized controlled trial (RCT), in which the intervention arm used a mobile app (eMOM), we conducted a mixed methods study that included an investigation of eMOM log files, semistructured interviews on self-discovery, and an examination of questionnaires assessing motivation (Treatment Self-Regulation Questionnaire and Perceived Competence Scale), depression (Edinburgh Postnatal Depression Scale), technology use and acceptance (Unified Theory of Acceptance of Use of Technology questionnaire), and usability (modified Software Usability Measurement Inventory). Additionally, we monitored participants’ stress levels using wearable electrocardiographic devices (FirstBeat Bodyguard 2). A total of 148 individuals participated in the original RCTs, with 76 in the intervention arm and 72 in the control arm. From the intervention arm, 18 participants were randomly selected for interviews in this study.

**Results:**

Results show that the use rate of eMOM was high, and novel visualization supported self-discovery in persons with GDM. Most participants (17/18, 94%) indicated that the eMOM app helped to find the associations between their daily activities and glucose levels. Especially having nutrition visualized together with glucose was highly appreciated. Participants also reported learning about the associations between physical activity and glucose levels. No differences were observed between the intervention and control arms in autonomous motivation, depression, or stress. Furthermore, there were no correlations between improved clinical outcomes and changes in motivation. Accessibility and usability ratings were consistently high throughout the intervention.

**Conclusions:**

The eMOM mobile app combining data from continuous glucose monitor, food diary, and physical activity tracker supports maternal self-discovery related to GDM without contributing to depression or adding extra stress. This encourages the use of such mobile apps in maternity care. Notably, motivational factors did not correlate with the positive outcomes observed in our prior RCT, suggesting that self-discovery has a greater impact on clinical results.

**Trial Registration:**

ClinicalTrials.gov NCT04714762; https://www.clinicaltrials.gov/study/NCT04714762

## Introduction

Gestational diabetes mellitus (GDM) is an increasing issue in maternal health care as it affects approximately 14% of pregnant individuals globally [1]. GDM increases the risk of developing type 2 diabetes and cardiovascular diseases [2,3], and also predisposes the child to adulthood obesity and type 2 diabetes [4].

In order to respond to the increasing demand for treatment, digital health is a topic of growing interest, as it provides innovative approaches to support the clinical management of various conditions. Mobile apps and wearable devices provide an opportunity for continuous tracking of numerous parameters for self-management of noncommunicable diseases. Diabetes apps have been shown to improve patient self-management in type 1 and 2 diabetes [5-8], while also being cost-effective [9]. Recent studies have provided promising findings on telemedicine in the management of GDM [10-16], but the effective interventions have involved significant effort from health care professionals regularly, which is not an optimal use of digital tools.

An emerging area of interest within digital health is the concept of self-discovery through self-tracking data. In diabetes management, this involves individuals using data on daily activities—such as nutrition, physical activity, sleep, and stress—to understand how these behaviors affect glucose levels. This concept, which has gained attention in the field of human-computer interaction [17-19], provides a valuable framework for understanding how self-tracking can enhance the self-management of diabetes.

To enhance the effectiveness of mobile apps designed for GDM management without the involvement of health care professionals, we developed an easy-to-use eMOM app that differs from the prior diabetes mobile apps in several aspects. First, it visualizes data from a continuous glucose monitor (CGM), physical activity sensor, and digital food diary in real time within the same app [20]. Second, the idea of eMOM is to teach a patient how their own lifestyle choices affect their glucose levels without extra help from health care personnel. Third, eMOM is used periodically, 1 week a month, to avoid getting tired of digital devices. The recent results of our randomized controlled trial (RCT) demonstrated that individuals with GDM who used eMOM improved their fasting glucose levels, enhanced physical activity, increased vegetable intake, and resulted in less weight gain during pregnancy [21]. Additionally, the incidence of newborns with macrosomia was lower among the individuals using eMOM [21]. Many of these improved clinical outcomes were associated with the use of eMOM, suggesting that eMOM supported self-management of persons with GDM.

The aim of this study is to evaluate how the use of eMOM affects the underlying maternal learning processes, that is, self-discovery, in the context of GDM self-management. We will also examine whether eMOM affects autonomous motivation for GDM self-management or psychological well-being. Additionally, we will assess whether there is an association between motivational factors and improved clinical findings in the RCT study. Finally, we will evaluate the acceptance and usability of the eMOM app.

## Methods

### Design

This study is a secondary analysis of eMOM GDM RCT [20], where the intervention arm used the eMOM app with a CGM and activity bracelet for 1 week per month and a digital food diary for 3 days of this week (referred to as the ‘app week’ later in this paper), from gestational weeks 24-28 to delivery. The eMOM GDM study was conducted between February 2021 and December 2022 in Helsinki, Finland. Patients were recruited from antenatal clinics in the Helsinki metropolitan area by research nurses.

Participants eligible for the study were persons aged 18-45 years with a diagnosis of gestational diabetes at gestational weeks 24-28. Individuals were excluded from the study if they had preexisting type 1 or type 2 diabetes, were using medications that affect glucose metabolism (such as continuous corticosteroid therapy or metformin), had multiple pregnancies, physical disabilities, current substance abuse, severe psychiatric disorders that could complicate participation, or significant difficulties in cooperating, such as inadequate Finnish language skills.

Participants were randomized 1:1 into intervention and control arms. The intervention arm received standard clinical antenatal care in addition to the eMOM app, while the control arm received only standard clinical care. Standard clinical care consisted of periodic visits with a nurse and a doctor in municipal maternal clinics, as offered for all pregnant individuals in Finland. A regular care protocol for low-risk pregnancies includes 9-10 visits with a nurse and 2 visits with a doctor. After being diagnosed with GDM, individuals receive guidance on diet, physical activity, and self-monitoring of blood glucose with electronic capillary glucose meters [22].

### Ethical Considerations

The study was conducted in accordance with the Declaration of Helsinki and received approval from the ethics committee of Helsinki University Hospital (approval no. HUS/2165/2018). All participants enrolled voluntarily, provided written informed consent, and were informed of their right to withdraw from the study at any time without consequence. No financial compensation was offered for participation. All data were pseudonymized and nonidentifiable to protect participant confidentiality.

### eMOM App

**Figure 1:**
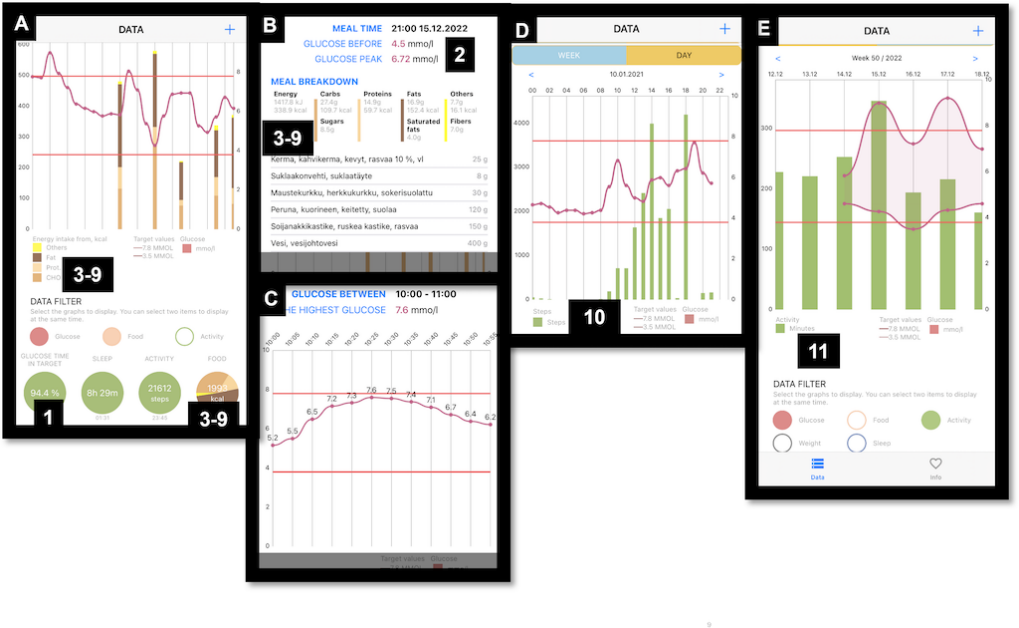
(A) Day view with nutrition and glucose filters selected, (B) detailed nutrition view, (C) detailed glucose view, (D) day view with physical activity (steps), and (E) week view with glucose and physical activity (minutes) selected. The following outcomes were visible to the user through the eMOM: (1) CGM glucose time in range (3.9-7.8 mmol/l), (2) postprandial increase of continuous glucose in 2 hours (mmol/l), (3) energy intake (kJ/day), (4) intake of carbohydrate (g/day), (5) intake of total fat (g/day), (6) intake of saturated fat (g/day), (7) intake of fiber (g/day), (8) intake of protein (g/day), (9) intake of sucrose (g/day), (10) number of walking steps (steps/day), and (11) duration of physical activity (minutes/day). CGM: continuous glucose monitor. Adapted from Kytö et al [21]. Screenshots (A-E) from the eMOM (Copyright: Fujitsu Finland).

The eMOM mobile app was developed to visually represent data from a tissue CGM (Medtronic Guardian Connect with Enlite Sensor) and an activity bracelet (Garmin Vivosmart 3) together with a digital food diary. The information from the sensors and food diary is updated every 10 minutes to eMOM. In our previous study [23], physical activity was measured with 3 different devices (activity bracelet, hip-worn sensor, and electrocardiography sensor). Most participants preferred the bracelet to hip sensor, so it was selected as an activity measurement tool for this study. Screenshots from the eMOM app are presented in Figure 1. For displaying the data, the eMOM app has 2 main views: a day view (Figures 1A and 1D) and a week view (Figure 1E). Nutrition intake from each energy-yielding nutrient is visualized as stacked bars (Figure 1A). More detailed information (recorded food items and nutrient intake in grams) can be accessed (Figure 1B) by tapping the stacked bar (Figure 1A). The eMOM app shows detailed glucose values (Figure 1C) when tapping the glucose curve on the screen (Figures 1A and 1D).

### Interactions With eMOM

The interactions with eMOM were collected in log files to determine how the participants used eMOM during the study. Each row in the log file included a pseudonymized user ID, timestamp, used view (day view, week view, detailed nutrition view, detailed glucose view, or info view), and filter used in day view (glucose, nutrition, and physical activity) or in week view (glucose, nutrition, physical activity, weight, and sleep).

### Questionnaires and Interviews

The background questionnaire covered experience with self-tracking at baseline (gestational weeks 24-28). Other background information, such as age, BMI, parity, and previous GDM status, were collected from hospital registries.

Maternal learning and self-discovery were assessed through interviews conducted with randomly selected participants from the intervention arm. These interviews took place after participants had been using the app for approximately 2 months. Interviews were semistructured and carried out remotely over videoconferencing software. They were audio or video recorded depending on the participant’s preference. They lasted from 30 to 56 minutes; the mean duration was 40 (SD 8.5) minutes. The main interview questions were, “Have you learned something while using the eMOM app? If yes, what?” “Has the eMOM helped you to make changes in your lifestyle? If yes, how?” “What features of the eMOM were most useful for you?” “How would you develop the app?” The interview script is provided in Multimedia Appendix 1.

To assess maternal motivation, we used 2 questionnaires based on the self-determination theory: the Treatment Self-Regulation Questionnaire (TSRQ) and the Perceived Competence Scale (PCS) for diabetes [24]. The TSRQ questionnaire assesses levels of autonomous versus controlled motivation, while the PCS measures perceived competence. Both questionnaires were administered at baseline (gestational weeks 24-28) and again between gestational weeks 35-37. Details of the questionnaires and scoring methods are available in Multimedia Appendix 2.

The maternal depression was measured with the Edinburgh Postnatal Depression Scale [25] at baseline (gestational weeks 24-28) and again at gestational weeks 35-37. The scale consists of 10 items, each scored from 0 to 3, yielding a total score range of 0 to 30. A score of 10 or higher is commonly used as the threshold to indicate possible depression [25].

The acceptance of eMOM was measured with the Unified Theory of Acceptance and Use of Technology questionnaire [26] after each app week (once a month), and the usability of the eMOM app was measured once, 4 weeks after taking the app into use with a questionnaire adapted from the Software Usability Measurement Inventory questionnaire [27].

### Stress Measurement

The Firstbeat Bodyguard 2, a chest-mounted sensor by Firstbeat Technologies, tracks heart rate variability (HRV) and physical activity using 3D acceleration over a continuous 3-day period. It uses 2 clinical-grade electrocardiographic electrodes for attachment and boasts a high accuracy in HRV measurement, with less than a 3-millisecond error and over 99.9% detection accuracy compared to standard electrocardiographic devices [28]. Additionally, Firstbeat’s Lifestyle Assessment software analyzes the HRV and motion data to evaluate stress and recovery levels, a methodology supported by extensive research validation [29,30]. Both the intervention and control arms used the Firstbeat sensor during the baseline period (gestational weeks 24-28) and again in the third trimester (gestational weeks 35-37). The sensor was worn continuously for 3 days during each phase.

### Analysis

The interviews underwent transcription, and 2 researchers (HY and SM) familiarized themselves with the content by reviewing the transcripts. The subsequent analysis followed the framework method, a recommended approach for multidisciplinary health research [31]. We used a combination of deductive and inductive approaches, where we had a set of initial codes (self-tracking of glucose, self-tracking of nutrition, and self-tracking of physical activity) at the beginning of the coding and used new codes when emerged from the gathered data. These new codes were combined into broader categories. The coding was performed by HY using ATLAS.ti software, which enabled charting of resulting themes and participants’ responses. This matrix presentation enabled us to analyze patterns, similarities, and differences in responses within each theme. Coding and the resulting themes were reviewed by 2 additional researchers (SM and MK). Quotes featured in the results were translated into English using an intelligent verbatim technique, which involves the removal of filler words like “er” during the translation process.

The log files were examined by computing the frequency and timing of interactions with the eMOM platform. Finally, we used triangulation across these various data sources (log files from eMOM, interviews, and questionnaires) to gain a comprehensive understanding of how self-tracking with eMOM supported self-discovery.

For comparing the difference between the arms, we performed a 2-sided *t* test for continuous outcomes, setting the significance threshold at 5%. For binary outcomes, we applied a chi-square test, resorting to the Fisher exact test when expected frequencies fell below 10. The difference between app weeks in the acceptance of eMOM was evaluated using repeated-measures ANOVA.

In analyzing the correlation between motivation and primary outcome from Kytö et al [21], we examined the linear relationship (Pearson correlation). For the binary outcomes, we used logistic regression.

## Results

### Participants

In total, 148 participants with GDM participated in the study. Out of 76 persons in the intervention arm who used the eMOM app, 63 continued until delivery. The main reason for dropout was the need to start GDM medication. In total, 18 participants from the intervention arm were randomly selected for user experience interviews for this study. The log files were collected from all the participants in the intervention arm. Baseline characteristics are presented in Table 1. Participants were familiar with using mobile apps and activity trackers and had a good conception of the significance of different nutrients for the diet, as shown in Table 1. No significant differences were observed between the arms at the baseline.

**Table 1:** Background characteristics.

		Interviews (n=18)	Intervention arm (n=76)	Control arm (n=72)		Difference between intervention and control arm (95% CI)
Age (years), mean (SD)		32.8 (3.7)	34.2 (4.1)	34 (3.9)		0.2 (–1.1 to 1.5)
BMI, mean (SD)		27.5 (5.1)	27.5 (5.5)	26.7 (4.5)		0.8 (–0.8 to 2.4)
Parity—primiparous, n (%)		10 (56)	38 (50)	37 (51)		–1% (–18% to 15%)
Early GDM^a^, n (%)		7 (38)	37 (49)	35 (49)		0% (–16% to 16%)
GDM status (previous GDM), n (%)		2 (11)	17 (22)	18 (25)^b^		–3% (–17% to 11%)
Gestational week at enrollment, mean (SD)		27 (1.8)	26.5 (1.7)	26.3 (1.7)		0.2 (0.4 to –0.7)
Weeks in the study at the interview, mean (SD)		9.3 (2.6)	—^c^	—		—
“**I am used to use mobile apps,” n (%)^d^**						
	1	0 (0)	1 (2)	4 (6)	—	
	2	0 (0)	0 (0)	1 (2)	—	
	3	0 (0)	0 (0)	0 (0)	—	
	4	2 (15)	8 (13)	5 (8)	—	
	5	11 (85)	55 (86)	54 (84)	—	
“**I am used to use activity trackers (like Fitbit and Polar),” n (%)^d^**						
	1	1 (8)	11 (17)	11 (18)	—	
	2	0 (0)	3 (5)	4 (6)	—	
	3	1 (8)	4 (6)	1 (2)	—	
	4	3 (23)	18 (28)	20 (32)	—	
	5	6 (46)	28 (44)	27 (43)	—	
“**I am familiar with measuring my blood glucose,” n (%)^d^**						
	1	2 (15)	10 (16)	3 (5)	—	
	2	3 (23)	3 (5)	6 (10)	—	
	3	2 (15)	4 (6)	1 (2)	—	
	4	3 (31)	21 (33)	19 (30)	—	
	5	3 (38)	25 (41)	34 (54)	—	
“**I am familiar with keeping a food diary,” n (%)^d^**						
	1	1 (8)	7 (11)	5 (8)	—	
	2	0 (0)	8 (13)	5 (8)	—	
	3	0 (0)	4 (6)	2 (3)	—	
	4	8 (62)	48 (48)	34 (54)	—	
	5	4 (31)	14 (22)	17 (27)	—	
“**I know the significance of different nutrients, fiber, and fat for the diet,” n (%)^d^**						
	1	0 (0)	2 (3)	1 (2)	—	
	2	0 (0)	0 (0)	3 (5)	—	
	3	0 (0)	2 (3)	2 (3)	—	
	4	6 (46)	28 (44)	25 (40)	—	
	5	7 (54)	32 (50)	32 (51)	—	
Autonomous motivation (TSRQ^e^), mean (SD)^f^		4.86 (0.51)	4.85 (0.51)^b^	4.68 (0.52)^g^		0.17 (–0.01 to 0.34)
Perceived competence (PCS^h^), mean (SD)^f^		5.71 (1.18)	5.60 (0.97)^b^	5.49 (1.18)^g^		0.11 (–0.25 to 0.47)
Elevated depressive symptoms (EPDS^i^ points>10), n (%)		3 (17)	12/71 (17)^j^	10 (14)		3% (–8.8% to 14.8%)
Stress percentage		53.5 (13.2)^j^	56.1 (13)^k^	57.5 (11.7)^l^		–1.4 (–6 to 3.2)

^a^GDM: gestational diabetes mellitus.

^b^Result missing from 1 participant.

^c^Not applicable.

^d^The Likert scale was from 1=strongly disagree to 5=strongly agree.

^e^TSRQ: Treatment Self-Regulation Questionnaire.

^f^The scale was from 1=low to 7=high.

^g^Result missing from 4 participants.

^h^PCS: Perceived Competence Scale.

^i^EPDS: Edinburgh Postnatal Depression Scale.

^j^Result missing from 1 participant.

^k^Result missing from 2 participants.

^l^Result missing from 3 participants.

### eMOM Use

Users interacted with the eMOM app most frequently during the morning hours (7-10 AM) and the evening hours (7-10 PM). Interaction levels were lowest at night (12-5 AM), increased sharply around 6 AM, peaked at 9 AM, and remained relatively steady throughout the day with a secondary rise in the evening.

During the first week of use, participants interacted with the app an average of 18.7 (95% CI 17-20.4) times per day. In the following application weeks, daily interactions ranged between 10 and 12 times. Between active usage weeks, the interaction frequency dropped significantly to 1-3 times per day.

**Figure 2:**
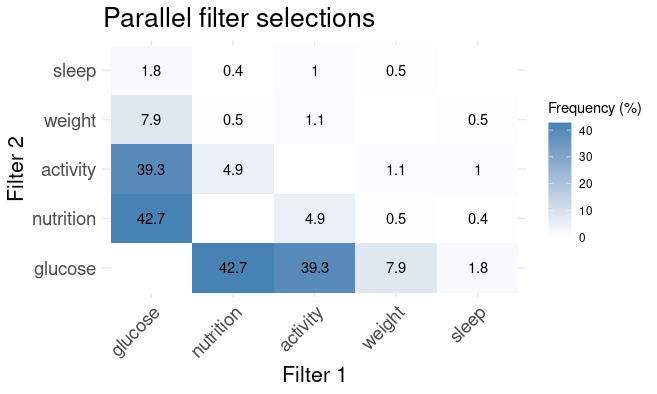
Parallel filter selections.

Regarding app usage patterns, the day view was by far the most commonly used feature, accounting for 65% of total interactions (Table 2). This was followed by the week view (18%), detailed glucose view (10%), information section (4%), and detailed nutrition view (3%). When viewing data, users most frequently applied the glucose filter (65% of the time), followed by the nutrition and physical activity filters (each 15%), while the weight and sleep filters were used only 3% of the time (Table 3). Glucose was mostly viewed together with nutrition and physical activity, as shown in Figure 2.

**Table 2:** Usage of different views.

View	Frequency (%)
Day view	65
Week view	18
Detailed glucose view	10
Information section	4
Detailed nutrition view	3

**Table 3:** Usage of different filters.

Filter	Frequency (%)
Glucose	65
Nutrition	15
Physical activity	15
Weight	3
Sleep	3

### Self-Discovery

The majority of interviewed participants (17/18, 94%) reported that the eMOM app facilitated their understanding of the connections between their daily activities and glucose levels. Primarily, participants found their learning experiences centered around nutrition (15/18, 83%) or physical activity (8/18, 44%). Seven (39%) participants acknowledged the combined influence of both nutrition and activity on their glucose levels. The interviews highlighted various methods of learning, such as experimentation, comparison, and observation.

Regarding self-discovery, 335 codes and 22 categories emerged from the interview data. These categories were then combined into main themes, which we present in a condensed form in the following sections. We have gathered all the supportive features of eMOM discussed in the interviews, along with direct comments from participants, and organized them into Table 4.

**Table 4:** Supporting features of eMOM identified through interviews.

Feature		How eMOM supports		Comments from the interviews	
**Nutrition**					
	Experimenting with different foods		The detailed nutrition view displays the specific foods in each meal and provides the breakdown of various nutrients, including the amount of carbohydrates, proteins, and fats, in grams.		“I switched from refined grains to whole grains, and it [glucose] has stayed pretty stable with that change.” [Participant 15] “If you consume, for example, a lot of carbohydrates in a meal, the effects show up immediately.” [Participant 18] “—It raised my blood sugar too high, or very close to the limit. So, I started to reduce my carbohydrate intake, which helped me find a balance. I realized that I should eat a small, protein-rich breakfast to kickstart my day. Then, after a couple of hours, I can have another meal with carbohydrates, and that keeps my blood sugar stable.” [Participant 8] “I learned that I can't eat fruits in the morning because they're too high in sugar after a night's fasting.” [Participant 3]
	Meal rhythm		The nutrition filter displays meals as bar graphs on a daily timeline, allowing users to view them alongside the glucose curve.		“I hadn't realized that I was going way too long between meals. Like, you should eat more often. And by looking from the app I have noticed that if I don't eat regularly enough, it [glucose] starts to rise.” [Participant 10] “Having a proper meal later in the evening has the biggest effect. I mean, if I haven't eaten enough, my glucose levels spike.” [Participant 11] “I experimented with a variety of foods and discovered that eating a chocolate bar alone in the middle of the day spikes my blood sugar, while having it as a dessert after lunch does not necessarily have the same effect.” [Participant 12]
	Portion sizes		The nutrition filter displays the calorie count for each meal. Portion sizes are visually represented with a bar graph, making it easier to see the proportion of meals throughout the day.		“Some restaurant meals etc. are so big that the blood sugar rises very high for a very long time. It was a very useful fact for me, as I immediately quit eating those.” [Participant 12] “I gained quite a good understanding of the number of calories in my meals and their variation. Since it is presented as bar graphs, it helps to get some insight into what changes I should make to my meal habits in the future.” [Participant 17]
**Activity**					
	General activity level		Activity filter shows the number of steps and active minutes and can be compared to the glucose curve.		“On February 19th, I was quite active, which kept my glucose levels within their normal range. However, on February 20th, I wasn't very active, and my glucose levels immediately rose above the threshold values. I’ve been comparing them in this way.” [Participant 2] “I have noticed that when I'm more active, the glucose level decreases faster.” [Participant 16] “By selecting both the activity and glucose filters, you can see the number of steps, and it becomes clear that, for example, taking a longer walk has an immediate effect on glucose levels.” [Participant 1]
	Physical activity after a meal		The activity filter displays the number of steps per hour using bar graphs. These data can be viewed alongside the glucose curve, allowing users to observe the effect of physical activity on glucose levels on an hourly basis.		“The other day I took a longer walk on a forest track after a meal and noticed that the glucose level didn´t rise as high as it normally would after a meal.” [Participant 3] “I learned very concretely that if I take kids to football practice right after a meal, glucose starts to decrease even after a bigger meal. But if I sit at a brunch table with my friends, it [glucose] rises very high very easily. This was very useful information, I think.” [Participant 12] “I noticed that if I, for example, take a short walk after eating, the glucose curve starts decreasing more.” [Participant 8]
Other supporting features of data visualization		eMOM integrates nutrition and activity data in one place, offering a clearer view of glucose variation, and displaying target glucose values, which helps users track and manage their condition more effectively.		“The eMOM app gathered both nutrition and food data in the same place, so if you wanted to do some kind of learning analysis about your nutrition or activity, the eMOM app was definitely better [versus only CGM/Medtronic] since it shows the combined data.” [Participant 3] “CGM/Medtronic does not display the glucose target, like upper and lower bounds. I think that, for example, the variation in glucose level is more clearly presented in the eMOM app.” [Participant 6] “Having target values on glucose helps to see how much you exceed the target.” [Participant 8]	

### Learning Between Nutrition and Glucose

The majority of the participants (15/18, 83%) discovered the patterns of association between nutrition and glucose. Nutrition information was presented through stacked bar charts, with a possibility to be integrated with the glucose curve, enabling direct comparisons of how various meals impacted glucose levels. Participants engaged in experimentation and comparison, evaluating different foods and nutrients, mealtimes, and portion sizes.

As shown in Figure 2, glucose was mainly viewed alongside nutrition (43% of the time). Table 4 illustrates user comments on how nutrition data has contributed to their self-discovery.

### Learning Between Physical Activity and Glucose

Although the activity was viewed with similar frequency as nutrition (see Table 3) and nearly as frequently in parallel with glucose visualization (39% of the time; Figure 2), it contributed less to self-discovery. However, almost half of the participants (8/18, 44%) noted a decrease in their glucose levels following exercise. Participants also assessed their activity levels on different days to explore the relationship between their activity—both active and inactive days—and their glucose levels on those days. Table 4 summarizes user comments on how activity data contributed to their self-discovery process.

### Learning Between Sleep and Glucose

Sleep data were found to be challenging to interpret, with a mere 3% viewing frequency (Table 3). Four participants reported discrepancies between the sleep data provided by the device and their own assessments of sleep duration and quality. One participant observed an association between a poorly slept night and elevated morning glucose level.

### Supporting Features of Learning in the eMOM App

The visualization of measurements emerged as a pivotal aspect of the learning process. Through observing the glucose curve, participants noticed fluctuations in glucose levels. When various filters (nutrition, activity, and sleep) were applied to the same graph alongside the glucose curve, participants could observe how different factors influenced glucose levels. The ability to view nutrition information in a quantitative format was also valued, and having a specific glucose target range was found to be important. Participants found the eMOM app more informative than Medtronic’s CGM in evaluating the overall picture of glucose balance. Table 4 summarizes the primary features used by individuals with GDM in the self-discovery process and how the eMOM app supported the identification of associations between these features and glucose levels.

### Temporal Progression of Learning

In the evenings, eMOM served as a tool for obtaining a comprehensive overview of the day’s events, as 1 participant described the following.

And then, at the end of the day, I looked at the eMOM application to see how the day had gone overall. I used it to get a summary.

Participant 9

Conversely, during the day, participants tended to rely on Medtronic for checking glucose levels, as it offered easier and quicker measurements due to its lower latency, updating every 5 minutes compared to eMOM's 10-minute intervals.

For most participants (n=10, 56%), the first week of using the app was found to be the most significant in terms of learning. Two participants expressed.

The most useful was the first application week; that's when I learned the most.

Participant 14

The most benefit came during those initial weeks, when I could really see how my diet affected me. Based on that, I was able to adjust it to suit myself better.

Participant 11

This reflects their strong interest in monitoring glucose levels during this period. As a result, interactions with eMOM were most frequent during this initial learning phase, as illustrated in Figure 2.

Periodic use of the eMOM app was found to be effective in maintaining motivation and sustaining interest in tracking activities. As 1 participant noted.

If it was available all the time, it probably wouldn't be used as much. Like, at the beginning, it is probably the most interesting, but when you learn to know how different things affect, then you no longer are interested in observing it.

Participant 15

### Inhibiting Features and Improvement Suggestions

Some participants (5/18, 28%) expressed a desire for enhanced informativeness by having all three filters (glucose, nutrition, and activity) integrated into the same graph.

A desire for additional support in interpreting data was expressed by 11 (61%) participants, particularly in understanding the impact of sleep data on glucose levels. Moreover, personalized recommendations regarding nutrition and more specific daily goals for macronutrients were requested. Preferences regarding support varied. While 1 participant mentioned seeking guidance from a nurse to interpret glucose data and 2 participants wished for assistance from health care personnel, others (8/18, 44%) would have been satisfied with suggestions or notifications from the app.

In this intervention, the activity bracelet tracked only steps and overall daily activity. Participants desired a broader range of sports tracking capabilities. For example, swimming is a preferred activity among pregnant persons, but Vivosmart 3 was not able to track swimming. Issues with activity tracking, such as inaccurate step counting during activities like walking with a stroller or counting knitting as steps, led to a sense of distrust in the accuracy of activity data.

One participant expressed a desire for guidance on physical activity, including recommendations on duration and types of exercises to meet daily activity goals. Some also mentioned that motivation toward physical activity decreased as pregnancy progressed and exercise levels decreased.

Furthermore, some participants noted that when their glucose levels remained within target ranges, their motivation to use the app and adhere to a healthy diet diminished. As 1 participant articulated.

My glucose values have stayed quite good, but if I would have had more variation in glucose values it might have been easier to see associations between things. But since my glucose values have been on target, I haven't really made any changes to my diet or other things I otherwise might have done.

Participant 14

### The Effect of eMOM on Motivation, Depression, and Stress

Autonomous motivation (measured with TSRQ) decreased in both arms: –0.11 (SD 0.38) in the control arm and –0.18 (SD 0.32) in the intervention arm between the baseline and gestational weeks 35-37. The between-arm difference was not statistically significant for the change of TSRQ (difference: –0.09; t_97_=1.15; *P*=.25) nor for the change of perceived competence (difference: –0.27; t_97_=1.21; *P*=.23). There was no statistically significant difference between the arms in the incidence of Edinburgh Postnatal Depression Scale scores greater than 10 (*χ*^2^_1_=0.10; *P*=.75). The summary of the results on the motivation is presented in Table 5.

**Table 5:** The effect of eMOM on autonomous motivation, competence, EPDS^a^, and stress percentages.

	Control arm		Intervention arm		Statistics		
	n	Mean (SD)	n	Mean (SD)	Difference	*t* test (2-tailed)	*P* value
Change in autonomous motivation (TSRQ^b^)	48	–0.10 (0.41)	51	–0.19 (0.36)	–0.09	1.15 (97)	.25
Change in perceived competence (PCS^c^)	48	0.16 (1.03)	51	–0.11 (1.17)	–0.27	1.21 (97)	.23
Incidence of EPDS>10	52	5 (10)^d^	52	6 (12)^d^	1 (2)^d^	0.10 (1)^e^	.75
Change in stress percentage	51	1.4 (12.5)	58	4.1 (11.9)	2.7	1.18 (107)	.24

^a^EPDS: Edinburgh Postnatal Depression Scale.

^b^TSRQ: Treatment Self-Regulation Questionnaire.

^c^PCS: Perceived Competence Scale.

^d^n (%) values.

^e^Chi-square test.

### Correlations Between Motivation and Improved Clinical Outcomes

Correlations between autonomous motivation (TSRQ) and perceived competence (PCS) and improved primary and secondary clinical outcomes [21] in the intervention arm are shown in Table 6. No significant correlations were found in change of motivational factors and improved clinical outcomes.

**Table 6:** Correlations between autonomous motivation (TSRQ^a^) and perceived competence (PCS^b^) on clinical outcomes.

Outcome where the effect of the intervention was observed in Kytö [21]	Change in autonomous motivation				Change in perceived competence		
	n	Pearson coefficient (*r*)	*P* value	n		Pearson coefficient (*r*)	*P* value
Primary outcome: Change in fasting glucose from the baseline to 35-37 gestational weeks (mmol/L)	49	–0.02	.88	49		–0.05	.75
Mean morning capillary fasting glucose (mmol/L)	35	–0.11	.52	35		0.26	.12
Timing of medication start^c^	10	–0.20	.54	10		0.25	.43
Gestational weight gain from the baseline to delivery (kg)	49	0.07	.61	49		0.06	.67
Change in intake of vegetables (g/MJ)	36	–0.04	.79	36		0.24	.15
Change in duration of sedentary behavior (minute/day)	46	–0.09	.54	46		–0.14	.35
Change in duration of light-physical-activity (minute/day)	46	0.05	.74	46		0.06	.67
Macrosomia (>4 kg)^d^	49	1.26^e^	.44	49		–0.35^e^	.48

^a^TSRQ: Treatment Self-Regulation Questionnaire.

^b^PCS: Perceived Competence Scale.

^c^Only the baseline measurement of motivation available.

^d^Logistic regression.

^e^β coefficient.

### The Acceptance and Usability of eMOM

The acceptance of eMOM and wearable sensors was high over the whole intervention (see Table 7 for a subset of statements and Multimedia Appendix 3 for responses to all the statements). For example, participants found the eMOM and the sensors useful for supporting lifestyle changes and they perceived eMOM easy to use.

**Table 7:** Responses to a subset of statements in the UTAUT^a^ questionnaire^b^.

		App week 1 (n=67)		App week 2 (n=47)		App week 3 (n=26)		App week 4 (n=10)	
		Somewhat agreed, agreed, or totally agreed (Likert scale 5-7), n (%)		Somewhat agreed, agreed, or totally agreed (Likert scale 5-7), n (%)		Somewhat agreed, agreed, or totally agreed (Likert scale 5-7), n (%)		Somewhat agreed, agreed, or totally agreed (Likert scale 5-7), n (%)	
**Perceived usefulness**									
	I would find the eMOM app with the sensors useful for supporting healthier lifestyle.		65 (94)		45 (94)		26 (100)		10 (100)
	Using the eMOM app with the sensors enables me to improve my lifestyle more quickly.		64 (93)		45 (94)		25 (96)		10 (100)
	If I use the sensor, I will increase my chances of getting a concrete lifestyle improvement.		65 (94)		44 (92)		24 (92)		10 (100)
**Perceived ease of use**									
	My interaction with the sensor is clear and understandable.		62 (90)		44 (92)		26 (100)		10 (100)
	I find the eMOM app with the sensors easy to use.		61 (88)		46 (96)		24 (92)		10 (100)
**Attitude toward behavior and use**									
	Using the eMOM app with the sensors is a good idea.		68 (99)		46 (96)		24 (92)		10 (100)
	The eMOM app and the sensors make changing lifestyle more interesting.		67 (97)		46 (96)		25 (96)		10 (100)
**Intrinsic motivation**									
	Using the eMOM app and the sensors is fun.		53 (77)		31 (65)		20 (77)		9 (90)
	I like using the eMOM app and the sensors.		59 (86)		40 (83)		23 (88)		9 (90)
**Extrinsic motivation**									
	People who influence my behavior think that I should use the eMOM app and the sensors.		11 (16)		7 (15)		6 (23)		3 (30)
**Facilitation**									
	I have the resources necessary to use the eMOM app and the sensors.		65 (96)		44 (94)		26 (100)		10 (100)
	I have the knowledge necessary to use the eMOM app and the sensors.		65 (96)		46 (98)		26 (100)		10 (100)
	I could improve my lifestyle using the eMOM app and the sensors If there was no one around to help me with eMOM and sensors.		54 (81)		39 (83)		22 (85)		10 (100)
**Anxiety**									
	I feel apprehensive about using the system.		3 (4)		2 (4)		2 (8)		1 (10)
	The eMOM app and the sensors are somewhat intimidating to me.		1 (1)		0 (0)		0 (0)		0 (0)

^a^UTAUT: Unified Theory of Acceptance and Use of Technology.

^b^Participants responded to each statement in UTAUT questionnaire using a 7-point Likert scale ranging from 1=totally disagree to 7=totally agree.

**Figure 3:**
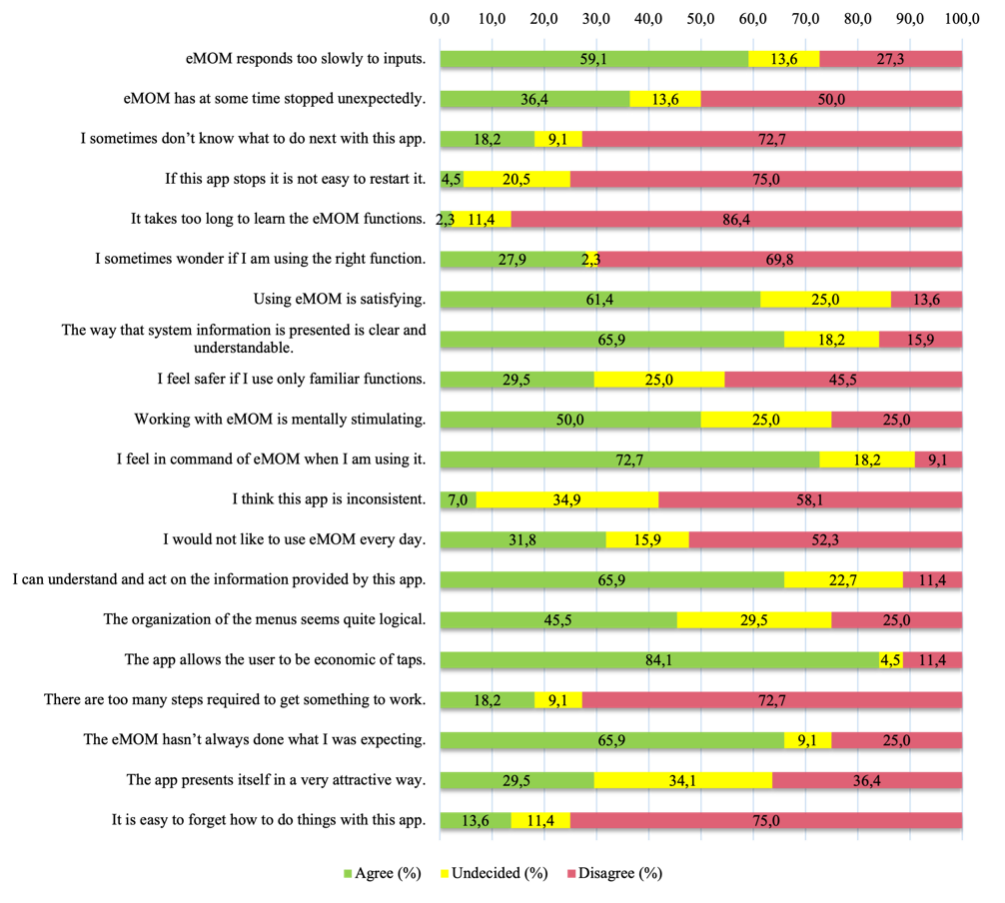
Responses to modified SUMI questionnaire (N=44). SUMI: Software Usability Measurement Inventory.

Responses to the usability questionnaire (modified from the Software Usability Measurement Inventory) were in line with responses to the Unified Theory of Acceptance and Use of Technology. For example, 86% (38/44) of participants disagreed with the statement “Learning the functions of the eMOM GDM application takes too long,” and 66% (29/44) agreed with the statement “I understand and know how to act based on the information provided by the application.” 59% (26/44) of participants agreed with the statement “eMOM answers too slowly to inputs,” which may have influenced the preference for Medtronic, which updates more quickly, during the day and eMOM more frequently in the evening. See Figure 3 for all the results of the usability questionnaire.

## Discussion

### Principal Findings

We found that a mobile app combining CGM, nutrition, and physical activity within the same app was highly used and supported self-discovery among persons with gestational diabetes. Providing pregnant persons with the opportunity to compare the effects of nutrition and physical activity on glucose levels facilitated their understanding of cause and effect. Almost all participants using the mobile app discovered associations between either nutrition or physical activity, and glucose levels. The majority found associations between nutrition and glucose, and nearly half of the participants identified associations between physical activity and glucose. There were no differences between the intervention and control arms in the change of perceived competence to manage GDM, autonomous motivation, depression, or stress scores. Additionally, autonomous motivation did not influence the improved clinical outcomes observed in our previous RCT [21]. Acceptance and usability remained high throughout the entire study period [21].

### Self-Discovery

The impact of an app that comprehensively integrates data in a single platform (including the automatic collection of activity and continuous glucose data) on self-discovery has not been previously studied in the context of gestational diabetes. Our study demonstrates that participants gained valuable insights into the associations between nutrition and glucose levels, facilitated by the eMOM app’s integrated data display. This comprehensive platform, which automatically collects activity and continuous glucose data, enabled users to observe the real-time impacts of their dietary choices on glucose levels. The ability to view glucose curves alongside nutritional details in a single window simplified the interpretation of meal timing and its effects, helping users better regulate their meal rhythms. Participants found value in comparing different foods and portion sizes, highlighting the importance of detailed nutritional information over simple carbohydrate tracking. These findings align with our previous work with various sensors, but without the eMOM app [23], where participants noted causal relationships between nutrition and glucose levels.

Learning the associations between physical activity and glucose proved to be somewhat more challenging than understanding the associations between nutrition and glucose. However, in this study, the associations between physical activity and glucose were more prominent compared to our study without the eMOM app [23]. The app’s parallel visualization of activity and glucose data may explain this improvement, as users could more easily observe associations between their physical activity and glucose changes. However, selecting features related to physical activity is challenging during pregnancy, as it complicates activity tracking. Activity bracelets typically track activity based on heart rate, which physiologically increases during pregnancy [32]. Currently, there are no activity bracelets designed specifically for pregnancy, which would account for these physical changes. While walking is a common form of physical activity during pregnancy [33,34], it may become difficult in the later stages. Swimming, water running, and weightlifting are preferred alternatives among pregnant individuals [33]. Therefore, it is essential to be able to track these alternative forms of physical activity. It is also worth noting that as pregnancy progresses, there is a general decline in physical activity levels [35,36]. Consequently, the relevance of physical activity in the management of GDM diminishes as pregnancy approaches its later stages. Similar challenges in physical activity tracking emerged in a study by Årsand et al [37] on type 2 diabetes, where patients expressed a desire for a broader range of trackable sports beyond just counting steps.

The eMOM app also provided data on sleep patterns, although interpreting this information proved challenging. Only 1 participant reported observing a potential connection between sleep and glucose levels, noting that during the flu accompanied by poor sleep, her glucose levels were higher. However, this association remains uncertain, as the flu itself can affect glucose levels. These results highlight the difficulty in finding associations between sleep patterns and glucose levels. Additionally, there is limited evidence on the effectiveness of wearable sensors on sleep during pregnancy [38]. Further investigation is needed in this area.

Recent research has explored ways to enhance the use of self-tracking data in diabetes self-management, although focusing primarily on type 1 and 2 diabetes [18,39-43]. However, self-management support is also crucial for individuals diagnosed with GDM, especially immediately following diagnosis, when adapting to the condition can be challenging [44,45]. Many individuals with GDM rely on trial-and-error learning [44-48], as seen in studies like that of Ibrahim et al [44], where participants expressed a desire to have all their self-monitoring tools gathered in one place to aid in sense-making. The recent review by Safiee et al [49] further supports this, showing that telemedicine interventions in GDM management improved self-management skills, particularly around understanding nutrition's impact on glucose. However, all prior reviewed interventions lacked CGM. Capillary glucose measurements capture only single-point readings, missing the full-day glucose fluctuations, including nocturnal hyperglycemia, which has been linked to a higher likelihood of large-for-gestational-age babies [50]. In contrast, the eMOM app provided a more comprehensive view of glucose levels, allowing participants to track changes over time. It is, however, worth noting that although CGMs have been found to be acceptable among persons with GDM [51-54], their use alone may not lead to improved glycemic control [54,55] or a reduction in macrosomia (birth weight > 4 kg) [53]. One contributing factor is the lack of clarity regarding the cause-and-effect relationships between lifestyle choices and glucose levels among persons diagnosed with GDM [45,47,56-58]. eMOM provides a tool for observing these causal relationships, thus enhancing the self-discovery process in managing GDM.

These findings emphasize the value of comprehensive data visualization tools in facilitating meaningful self-discovery in GDM management, offering both immediate feedback and clearer connections between daily behaviors and glucose outcomes.

### Motivation

While previous studies indicate a strong motivation among individuals with GDM to manage their condition [45,48,59], our study aimed to understand how eMOM influences motivation, with a particular focus on autonomous motivation and perceived competence—both key components of self-determination theory. Contrary to the findings of Williams et al [60], who demonstrated a significant correlation between improved glucose levels and autonomous motivation among type 2 diabetes patients, our findings suggest that the eMOM app did not lead to improved clinical outcomes through increased autonomous motivation. At the start of the intervention, our study participants in both the intervention and control arms were already highly motivated, leaving little room for further increases in autonomous motivation. Additionally, those using eMOM did not increase their perceived competence to manage GDM compared with the control arm. These findings suggest that the improved maternal clinical outcomes reported earlier [21] are primarily attributed to self-discovery rather than increased motivation. Thus, motivational interventions that are effective in type 2 diabetes management may not directly apply to GDM. Instead, educational approaches, such as the eMOM app, play a crucial role in helping persons with GDM learn how to manage their condition effectively.

### Strengths and Limitations

This study is the first to combine qualitative and quantitative approaches to investigate how comprehensive self-tracking influences self-discovery and motivation in managing GDM. The eMOM app offers a novel approach to GDM self-management by integrating CGM, nutrition tracking, and physical activity monitoring in real-time within a single app, without guidance from health care personnel, thus addressing a gap in previous GDM apps [61]. This integrated approach was also requested in our previous study, where participants used the same variable sensors and meters (CGM, physical activity, food diary, and sleep) as in this study, but separately in different apps and devices [23].

While some previous GDM apps have aimed to reduce reliance on health care personnel [15,16,62,63], none have incorporated the level of comprehensive and automated data tracking offered by the eMOM app. To our knowledge, only 1 earlier app, Habits-GDM by Yew et al [15], combined glucose data, a food diary, and physical activity tracking. It used capillary glucose measurements and monitored physical activity solely through step counts using a phone pedometer. The app also displayed only total calories and carbs, lacking detailed nutritional data. While Habits-GDM resulted in lower proportions of glucose levels above target and reduction in composed neonatal outcomes, none of the individual neonatal outcomes were statistically significant, and the intervention did not reduce gestational weight gain. Habits-GDM also included a messaging feature, allowing users to consult health care personnel and receive responses within 24 hours. However, the study did not report information on the app’s usage rates.

Similarly, MobiGuide by Peleg et al [62] combined capillary glucose measurements with step counts but lacked nutrition tracking. While it showed a significant reduction in blood pressure, it did not demonstrate any significant differences in glucose levels, insulin use, or neonatal outcomes. The Pregnant+ app by Borgen et al [63] allowed users to manually log physical activity, but none did, highlighting that manual logging alone was insufficient for meeting users’ needs.

As reported in the RCT study [21], eMOM use was consistently high. Most of the learning occurred, however, during the first application week. In the following application weeks, eMOM was mainly used to check if the glucose stayed within the limits. As noted by the participants, continuous availability of the sensors might lead to decreased motivation for tracking. Due to this periodicity, engagement with eMOM remained high during the application weeks, in contrast to the declining engagement observed in automatic self-tracking among people with type 2 and type 1 diabetes [64]. Participants continued to interact with eMOM even outside the designated application weeks, even when they did not have access to the activity bracelet or CGM. However, these interactions were significantly less compared to the application weeks. Throughout this study, the acceptance of eMOM and wearable sensors remained high, as indicated by questionnaires.

Previous research has suggested that extensive self-monitoring could lead to adverse psychological outcomes [65,66]. We found no evidence of an effect on depression or stress, as measured by Edinburgh Postnatal Depression Scale questionnaires and Firstbeat stress scores. This suggests that comprehensive tracking does not have a detrimental effect on maternal psychological well-being.

The most significant limitation concerning the self-discovery process was the inability to display nutrition, physical activity, and glucose data together in 1 graph. Participants expressed a need for more support in interpreting data and making informed choices. For example, the app could send notifications when glucose values exceed the target range and instruct users to review their recent meals.

Self-discovery’s impact on clinical outcomes was not objectively measured. However, the results of the interviews suggest that self-discovery had a positive effect on clinical outcomes. Another limitation is that individuals who required medication for GDM were excluded from the study. This may have introduced bias into the results of motivation, depression, and stress levels, as the analyses only included patients whose condition was managed well enough to avoid medication.

### Insights, Recommendations, and Future Directions

Implementing digital tools in clinical care requires patients to have health literacy. Low health literacy in patients with type 2 diabetes has been linked to lower engagement with mobile-based health interventions [67]. To be effective, information provided by these apps should be tailored to the health literacy levels of the target population [68]. In future research, eMOM should be evaluated in populations with varying levels of health literacy. Artificial intelligence–based solutions could assist users in understanding lifestyle improvements based on their health data.

Our findings suggest that an app presenting features in a quantifiable format is well-suited for the self-discovery process in GDM. Additionally, the limited number of features in GDM and the clear causal relationships between glucose and these features make this process easier to learn. Self-discovery in GDM exemplifies goal-driven tracking [69], particularly for monitoring nutrition and physical activity to maintain glucose levels within the recommended limits set by health care providers. We believe that our approach of integrating data from various sources in real-time could be beneficial for other noncommunicable diseases, such as type 2 diabetes, where the relationships between features and outcomes are relatively clear and learnable. Especially after diagnosis, when individuals are still in the learning phase of managing their condition, patients could benefit from a solution like ours.

Beyond the short-term effects on pregnancy outcomes, gestational diabetes also has long-lasting implications for maternal health [2,3]. Future research should investigate the extended impact of using the eMOM app, particularly in the postpartum period. Examining how this tool influences long-term health behaviors, psychological well-being, and the prevention of type 2 diabetes could offer valuable insights into its role in promoting sustained self-management beyond pregnancy.

### Conclusions

By visualizing CGM, nutrition, and activity in a single app in real-time, the eMOM app provides a promising tool to enhance self-discovery in GDM, without additional support from health care personnel. Our findings also suggest that eMOM’s impact is more related to self-discovery than to increased motivation. To support learning further, future work involves visualization of more than 1 feature impacting glucose at the same time and the use of artificial intelligence–based recommendations. In addition to GDM, this kind of mobile health solution could be used to support learning in other noncommunicable diseases, such as type 2 diabetes.

## Appendix

Multimedia Appendix 1 Semistructured interview questions.

Multimedia Appendix 2 TSRQ and PCS questionnaires. TSRQ: Treatment Self-Regulation Questionnaire; PCS: Perceived Competence Scale.

Multimedia Appendix 3 Responses to the UTAUT (Unified Theory of Acceptance and Use of Technology) questionnaire.

## Abbreviations

CGM: continuous glucose monitor
GDM: gestational diabetes mellitus
HRV: heart rate variability
PCS: Perceived Competence Scale
RCT: randomized controlled trial
TSRQ: Treatment Self-Regulation Questionnaire
