# Supplementary material for: The Effect of a Mobile App (eMOM) on Self-Discovery and Psychological Factors in Persons With Gestational Diabetes: Mixed Methods Study
Source: JMIR Mhealth Uhealth. 2025 Jun 4;13:e60855. doi: 10.2196/60855 (PMC12177430; doi:10.2196/60855)
Supplement: Multimedia Appendix 1 [file mhealth_v13i1e60855_app1.docx]

**Semi-structured Interview**

**Open-ended Interview Questions**

A. Questions about Learning and Provided Information

- - Have you learned something while using the eMOM app? If yes, what?
  - What do you think about the information provided by the application regarding blood sugar levels and what affects them?
  - What do you think about the information provided by the application regarding physical activity, and how does it affect your behavior/choices?
  - What do you think about the information provided by the application regarding sleep, and how does it affect your behavior/choices?
  - What do you think about the information provided by the application regarding nutrition, and how does it affect your behavior/choices?
  - What do you think about the information provided by the application regarding weight, and how does it affect your behavior/choices?
  - Have you learned anything while using the application? What?
  - Do certain areas stand out as more useful information?
  - What do you think about the amount of information provided - too much, too little? Why?
  - What about the way the information is presented? How would you describe it (clear, cluttered, readable, unclear, etc.)?

C. Questions about Problems and Challenges in Using the eMOM GDM Application

- - Could you describe a problem or challenge you have experienced using the eMOM GDM application?
  - Could you tell me about problems or challenges with measurements and sensors?
  - What about challenges in the daily use of the eMOM GDM application?

D. Questions about the Benefits of Using the eMOM GDM Application

- - What features of the eMOM were most useful for you?
  - Has the eMOM helped you to make changes in your lifestyle? If yes, how?
  - What benefits have you experienced from using the eMOM GDM application regarding your physical activity?
  - Regarding your sleep?
  - Regarding your eating and nutrition?
  - Regarding your blood sugar levels?
  - Regarding your weight management?
  - Has the eMOM GDM application helped you change your lifestyle? How?
  - Describe an event where the eMOM GDM application positively affected your actions? What about negatively?
  - Has the eMOM GDM application helped you make better choices? How?

E. Development Ideas

- - How would you develop the app?
  - Do you have any ideas on how the eMOM GDM application could be improved?
  - Could navigation be more intuitive or clearer for you? How?
  - What about the communication/visualization of information? How could it be improved in your opinion?

F. Conclusion

- - Do you have any questions?
  - Is there anything else you would like to add?

**General and Clarifying Questions** NOTE: These can be used at any time during the interview to obtain details or clarification, or to ensure the smooth flow of the interview.

- - How are you / How are things going?
  - Would you like to take a break?
  - Would you like to move on?
  - Could you clarify?
  - What made you think that way?
  - Could you show me how?
  - Why did you choose this?
  - How did you arrive at this?
  - How would you improve it?
