# Supplementary material for: The Effect of a Mobile App (eMOM) on Self-Discovery and Psychological Factors in Persons With Gestational Diabetes: Mixed Methods Study
Source: JMIR Mhealth Uhealth. 2025 Jun 4;13:e60855. doi: 10.2196/60855 (PMC12177430; doi:10.2196/60855)
Supplement: Multimedia Appendix 2 [file mhealth_v13i1e60855_app2.docx]

TSRQ and PCS questionnaires

| **Treatment Self-Regulation Questionnaire (TSRQ)**  Each item is rated on a scale from 1 to 7, where: 1 = Not at all true, 4 = Somewhat true, 7 = Very true. |  |
| --- | --- |

| The reason why I monitor my blood sugar is: |
| --- |
| Because people would be upset with me if I didn't. |
| Because I see it as a personal challenge. |
| Because I believe controlling diabetes will improve my health. |
| Because I would feel guilty if I didn't do what my healthcare professional instructed. |
| Because I want the healthcare staff to think I am a good patient. |
| Because I would feel bad about myself if I did not eat a healthy diet. |
| Because it is exciting to try to keep my blood sugar at a healthy level. |
| Because I don't want people to be disappointed in me. |
| The reason why I follow my diet and exercise regularly is: |
| Because people would be upset with me if I didn't. |
| Because I believe these are important things for staying healthy. |
| Because I would be ashamed of myself if I didn't. |
| Because it is easier to do what I am told than to think about it. |
| Because I have carefully thought about my diet and exercise and believe I am doing the right thing. |
| Because I want others to see that I can follow my diet and stay fit. |
| Because my healthcare professional instructed me to do so. |
| Because I feel that monitoring my diet and exercising are excellent things for me. |
| Because I would feel guilty if I didn't monitor my diet and exercise. |
| Because regular exercise and following my diet are choices I really want to make |
| Because it is a challenge to learn to live with diabetes. |

**Perceived Competence Scale (PCS)**

Each item is rated on a scale from 1 to 7, where: 1 = Not at all true, 4 = Somewhat true, 7 = Very true.

| I feel conﬁdent in my ability to manage my gestational diabetes. |
| --- |
| I am capable of handling my gestational diabetes now. |
| I am able to do my own routine diabetic care now. |
| I feel able to meet the challenge of controlling my gestational diabetes. |
